# Defining the Celiac Disease Transcriptome using Clinical Pathology Specimens Reveals Biologic Pathways and Supports Diagnosis

**DOI:** 10.1038/s41598-019-52733-1

**Published:** 2019-11-07

**Authors:** Nurit Loberman-Nachum, Katya Sosnovski, Ayelet Di Segni, Gilat Efroni, Tzipi Braun, Marina BenShoshan, Lait Anafi, Camila Avivi, Iris Barshack, Dror S. Shouval, Lee A. Denson, Amnon Amir, Ron Unger, Batia Weiss, Yael Haberman

**Affiliations:** 1 The Pediatric Gastroenterology Unit, The Edmond and Lily Safra Children’s Hospital, Sheba Medical Center, Tel-Hashomer, Israel; 2 Mina and Everard Goodman Faculty of Life Science, Bar-Ilan University, Ramat Gan, Israel; 3 Sackler Faculty of Medicine, Tel Aviv University, Tel Aviv, Israel; 4 Institute of Pathology, Sheba Medical Center, Tel Hashomer, Israel; 5 Cincinnati Children’s Hospital Medical Center, Department of Pediatrics, University of Cincinnati College of Medicine, Cincinnati, OH USA

**Keywords:** Gastrointestinal diseases, Gastroenterology

## Abstract

Celiac disease is provoked by gluten exposure, but the complete pathogenic process in the duodenum and the loss of tolerance to gluten is not well understood. We aimed to define the core celiac transcriptomic signature and pathologic pathways in pre-treatment formalin-fixed paraffin-embedded (FFPE) duodenum biopsies used for clinical diagnosis. We use mRNAseq to define pre-treatment diagnostic duodenum gene expression in 54 pediatric celiac patients and non-celiac controls, and we validate our key findings in two independent cohorts of 67 adults and pediatric participants that used fresh frozen biopsies. We further define similar and divergent genes and pathways in 177 small bowel Crohn disease patients and controls. We observe a marked suppression of mature epithelial metabolic functions in celiac patients, overlapping substantially with the Crohn disease signature. A marked adaptive immune response was noted for the up-regulated signature including interferon response, alpha-beta, and gamma-delta T-cells that overlapped to some extent with the Crohn disease signature. However, we also identified a celiac disease specific signature linked to increased cell proliferation, nuclear division, and cell cycle activity that was localized primarily to the epithelia as noted by *CCNB1* and *Ki67* staining. Lastly, we demonstrate the utility of the transcriptomic date to correctly classify disease or healthy states in the discovery and validation cohorts. Our data supplement recently published datasets providing insights into celiac pathogenesis using clinical pathology FFPE samples, and can stimulate new approaches to address this highly prevalent condition.

## Introduction

Celiac disease is a systemic immune mediated enteropathy triggered by dietary gluten in genetically susceptible individuals^1–3^. It is characterized by a broad range of clinical presentations, a specific serum autoantibody response, and variable damage to the small-intestinal mucosa. Globally, the prevalence of celiac disease is increasing. Studies comparing serum stored from 1948–54 and 1974–89 to recent samples from the USA showed 4 and 2 fold increase over approximately 50^4^ and 15^5^ years respectively, and a similar increase was noted over 20 years in Finland^6^. In light of the increasing prevalence and improved recognition, more complete understanding of the underlining pathogenesis may elaborate on preventive strategies in high risk individuals^7^, and on ways to improve treatment strategies.

Formalin-fixed, paraffin-embedded (FFPE) tissue samples stored in pathology archives represent an invaluable biobank for clinical research, and its use for transcriptomics was previously tested with good results^8^. To supplement recently published celiac disease mRNAseq transcriptomic studies^9,10^ done on fresh frozen biopsies, and to improve our understanding of celiac pathogenesis, we applied a standardized high throughput mRNA sequencing (RNAseq) approach on FFPE archived duodenum biopsies used for clinical diagnosis of active pre-treatment celiac disease and controls subjects (n = 54). Our cohort represent the largest celiac disease mucosal transcriptomic cohort to date^9–12^ (Table S1). We capture robust gene expression and pathways that are linked to celiac pathogenesis, which were validated independently in other cohorts^9,10^. Comparison of the celiac disease signature with our previously published Crohn disease signature showed similar and divergent pathways that can shed light on those intestinal inflammatory diseases, emphasizing the more unique signal for the increase in epithelial cell cycle and proliferation coupled with reduced epithelial mature metabolic function associated with epithelial de-differentiation in celiac disease.

## Methods

### Study design and participants

Newly diagnosed celiac disease and age-matched controls (Ctl) subjects on a gluten-containing diet were included in the study (Table 1). Celiac disease diagnosis was based on previously described algorithms^13^ including positive IgA autoantibodies against tissue transglutaminase (anti-TTG) and villous blunting consistent with Marsh 3 on duodenal biopsy. Histopathologic assessment was completed by a single pathologist. To mimic real-life referrals, we included subjects with abdominal pain, poor growth, or anemia as non-celiac controls. The Sheba Local Research Ethics Committee granted ethic approval for the study and waived the need for patients’ written informed consent for using archived formalin-fixed paraffin-embedded (FFPE) material. All methods were performed in accordance with the relevant guidelines and regulations.

**Table 1:** Patients’ demographics and disease characteristics.

	Non-Celiac discovery (n = 15)	Celiac discovery (n = 23)	Non-celiac validation (n = 6)	Celiac validation (n = 10)
Age (mean, SD)	8.3(4.3)	8.4(4.4)	7.7(4.8)	7.7(4.9)
Gender Male (n, %)	7 (47%)	11 (48%)	3 (50%)	5 (50%)
Abnormal TTG (n, %)	1 (7%)	23 (100%)	0(0%)	10 (100%)
TTG > 100U/ml (n, %)	—	15 (65%)	—	10 (100%)
Reported abdominal* pain (n, %)	13 (87%)	10 (43%)	4 (66%)	3 (30%)

TTG; tissue trans glutamines.

*From 15 non-Celiac discovery; 13 had endoscopy for abdominal pain, 1 for poor growth, and 1 for anemia. From 6 non-Celiac validation; 4 had endoscopy for abdominal pain, and 2 for poor growth.

### Duodenal RNA extraction and 3′ mRNA-seq Analysis

RNA was isolated from FFPE sections containing 4 pooled duodenal biopsies using the Qiagen AllPrep RNA/DNA FFPE Kit. Lexogen QuantSeq 3′ mRNA-Seq libraries^14^ and single-end 61 bp sequencing was performed^15^. Reads (mean of ~5.7 M per sample with 2.9 M Std. Deviation) were quantified by Kallisto v0.42.5^16^, using Gencode v24 as the reference genome with 2.1 M pseudo aligned mean reads per sample, after excluding one sample due to poor coverage. Estimated counts were normalized to Reads per Million (RPM). 54 RNAseq samples were included and stratified into specific clinical sub-groups (21 Ctl and 33 celiac disease) and randomly assigned to gender and age matched discovery and validation cohorts with a 10:3 ratio between discovery and validation cohorts respectively. 48/54 (89%) were obtained and stored in the pathology core during 2017 and RNA was extracted within one year (mean of 251 days), and 6 (4 celiac and 2 controls) were obtained before 2017, and were processed within 4 years. We included 14,778 protein-coding mRNA genes with RPM above 3 in 20% of the samples in our downstream analysis.

Differentially expressed genes were determined in GeneSpring® software using the discovery cohort (23 celiac and 15 controls) with fold change differences (FC) > = 1.5 and using the Benjamini–Hochberg false discovery rate correction (FDR, 0.05) and not on the validation cohort due to samples numbers constrains. Unsupervised hierarchical clustering using Euclidean distance metric and Ward’s linkage rule was used to test for groups of duodenal biopsies with similar patterns of gene expression in both the discovery and validation cohorts. Principal Component Analysis (PCA) was performed to summarize variation in gene expression between patients in discovery and validation cohorts. ToppGene^17^/ToppCluster^18^ and ClueGO^19^ platforms were used for functional annotation enrichment analyses and Cytoscape.v3.0.2^20^ for visualization. Two recent celiac transcriptomics studies were used for validation; FASTQ files from Leonard *et al*.^9^ were processed similarly, and the processed differentially expressed genes from Bragde *et al*.^10^ were used for downstream analyses and comparison. R package random Forest^21^ version 4.6.14 with out of box (OOB) estimate of error rate, and the Support Vector Machine (SVM) in GeneSpring® software were used to build a classification model to differentiate celiac from controls using the discovery cohort. Those models were used to test the accuracy of the classification in the independent validation cohort.

To compare between the celiac disease signature and our previous Crohn disease signature^22–24^ [GSE57945], we first confirmed that 92% (13,419 of 14,587) of the protein coding genes that passed the expression filtering criteria and were used for differential expression in the ileum in Crohn disease overlapped with the 14,778 protein-coding mRNA genes that passed expression filtering and were used for differential expression in the duodenum in the current study. We then used Venn diagrams that overlayed the core Crohn disease signature (derived from comparing Crohn disease and age/gender matched controls ileal biopsies) with the core celiac disease signature (derived from comparing Celiac disease and age/gender match controls duodenal biopsies) to test for similarities and differences, using only 1817/2160 of the Crohn disease differentially expressed genes that passed the current expression filtering criteria for downstream analyses.

### Quantitative PC (qPCR)

qPCR was performed on cDNA derived from FFPE extracted RNA as above. *SI* and *APOA1* mRNA expression was determined by SYBER Green Master Mix (Applied Biosystems) according to the manufacturer’s instructions assays, after normalization to GAPDH. Relative mRNA levels were expressed as fold change (Rq). Primers used are in Table S3.

### Immunohistochemistry

FFPE blocks were sectioned at 4μm and were processed by a fully automated protocol on a Benchmark Ultra staining module (Ventana Medical Systems Inc., USA). Briefly, after sections were dewaxed and rehydrated, a CC1 Standard Benchmark Ultra pretreatment for antigen retrieval was selected for *APOA4* (1:400, SIGMA, HPA001352, USA), *CCNB1* (1:100, SIGMA, HPA061448, USA),), *SI* (1:1000, SIGMA, HPA011897, USA), and *MKI67* (Ki67, 1:300, Thermo scientific, RM-9106-S,). *APOA4, SI*, and MKI67 were detected with UltraView and *CCNB1* was detected with OptiView DAB Detection Kits (Ventana Medical Systems Inc., USA). Sections were counterstained with Hematoxylin II (Ventana Medical Systems Inc., USA). The slides were dehydrated in graded ethanol (70%, 96%, and 100%). Before cover-slipping, sections were cleared in Xylene and mount with Entellan.

### Transcript profiling

Duodenal mRNAseq data sets were deposited into GEO [GSE131705], and we used our previously published Crohn disease transcriptomics [GSE57945].

## Results

### Decreased epithelial metabolic functions in celiac disease

**Figure 1:**
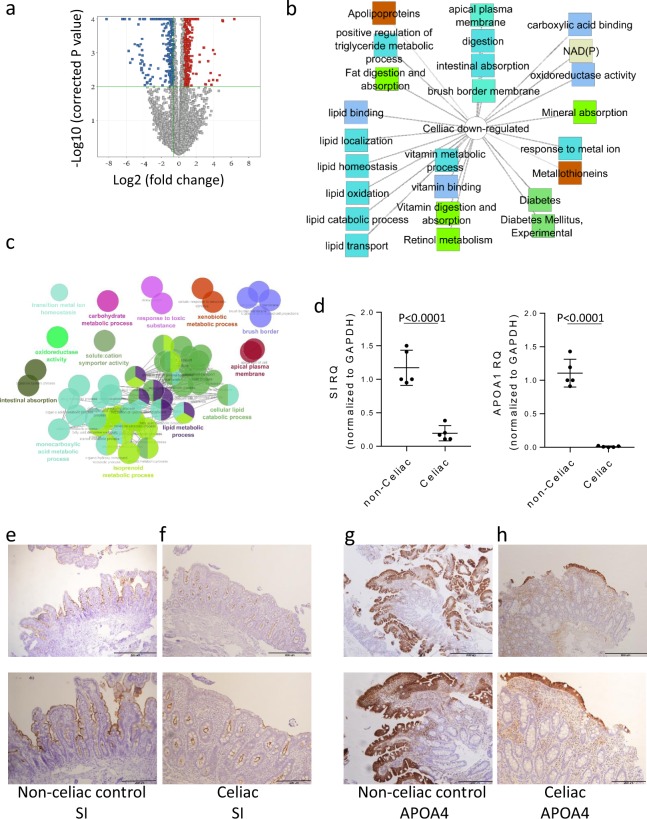
The core genes and pathways of newly diagnosed celiac disease emphasize reduced mature epithelial metabolic functions. (**a**) Volcano plot of the 878 differentially expressed genes between 23 celiac and 15 Ctl samples in the discovery cohort (FC ≥1.5 and FDR <0.05). Functional annotation enrichment analyses of the 354 down-regulated celiac core genes using ToppGene^17^/ToppCluster^18^ (**b**) and ClueGO^19^ (**c**), and visualized using Cytoscape^20^. In B, GO Biological Process, Cellular Component, and Molecular Function (blue), pathways (light green), gene family (brown), and disease (dark green). The full list of gene set enrichment results and P values are in Supplementary Dataset 1. (**d**) Relative quantification values (Rq, mean with SD) of *APOA1* and *SI* mRNA after *GAPDH* normalization for controls (n = 5) and celiac (n = 5) with two-tailed T-test p values. Immunohistochemistry stains of duodenal epithelia brush border *SI* (**e**, **f**) and cytoplasmic *APOA4* (**g**, **h**) for Ctl (**e**, **g**, n = 5) and celiac (**f**, **h**, n = 5). Ctl (**e**) subject show relatively higher brush border *SI* stain than celiac (**f**) that correlated with reads per million (RPM) values of 74 and 25 respectively. Ctl (**f**) subject show relatively higher *APOA4* stain than celiac (**h**) that correlated with RPM values of 194 and 19 respectively. Lower image is inlet of the upper image that were recorded at 20x magnification. Scale bar represents 200 and 500 microns.

We used archived clinical FFPE tissue. Our cohort included 54 children (mean age of 8 years), randomly assigned to 2:1 discovery and validation cohorts (Table 1). We specifically used the 3′UTR Lexogen platform^14^ that is designed for analyzing fragmented FFPE samples. Analyses of 3 FFPE and fresh paired biopsies obtained from the same endoscopic region showed correlation of ~0.8 (Figure S1) and was therefore supportive of this approach. We defined a core duodenal celiac gene expression signature composed of 878 genes (Fig. 1a) differentially expressed [FDR <0.05 and fold change (FC) ≥1.5] in comparison to controls (Ctl), using only the discovery cohort (Fig. 1 and Supplementary Dataset 1). Functional annotation enrichment analyses using ToppGene^17^ and ToppCluster^18^ mapped groups of related genes to biological processes^24^. *P* values for the top specific biological processes were obtained from ToppGene (Supplementary Dataset 1) and more detailed ToppCluster pathways analysis output is shown in Fig. 1b for the 354 down-regulated genes. The down-regulated celiac signature showed a robust decrease of epithelial lipid metabolic processes genes (*P* < 1.97E-11) and apolipoproteins (*P* < 5.07E-3), reduced vitamins metabolism and absorption (*P* < 4.29E-7), and lower oxidoreductase and NAD/P activities (*P* < 2.10E-6). Applying an independent ClueGO^19^ pipeline for functional annotation enrichment analyses is shown in Fig. 1c with similar results. Using quantitative PCR (qPCR) confirmed the reduction in sucrase-isomaltase (*SI*) and *APOA1* genes expression levels in celiac disease (Fig. 1d). Immunohistochemistry further demonstrated reduced epithelial abundance of *APOA4* protein that also showed a reduced expression in our dataset in the cytoplasm and *SI* in the brush border in active celiac disease patients (Fig. 1e–h) in comparison to non-celiac subjects. Importantly, a total of 403 genes were differentially expressed in at least in 2 of 3 recent RNAseq transcriptomic studies comparing active celiac and controls (current study, Bragde *et al*.^10^, and Leonard *et al*.^9^), and 85% (341/403) are within our core celiac signature (Figure S2 and Supplementary Dataset 1). Using ToppGene/ToppCluster confirmed the functional enrichment and the reduction of genes and pathways associated with lipid metabolism, and genes associated with oxidoreductase functions (Figure S3 and Supplementary Dataset 1).

### Increased cell cycle and nuclear division activity in celiac disease

**Figure 2:**
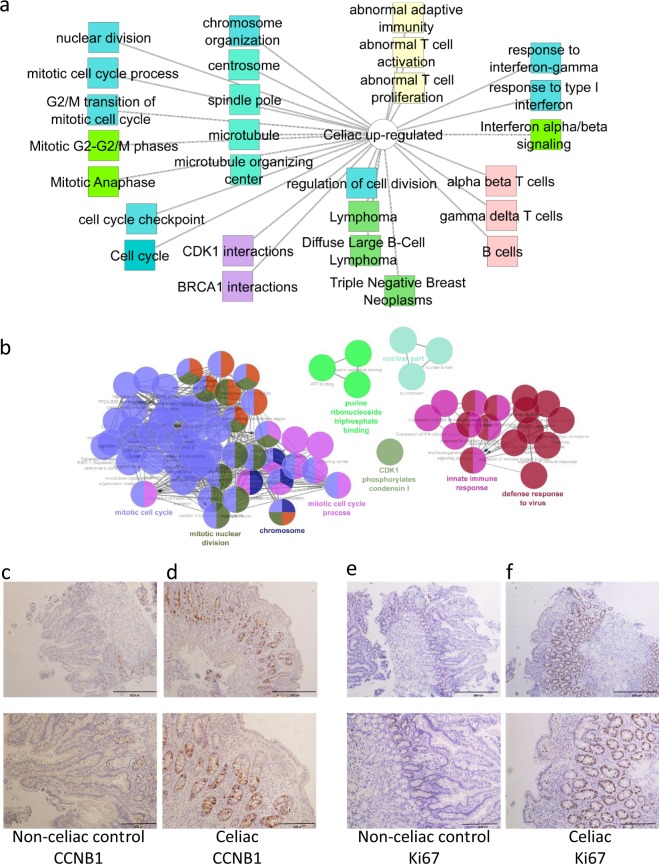
Increased cell cycle and nuclear division activity in celiac disease. 524 up-regulated celiac core genes using ToppGene^17^/ToppCluster^18^ (**a**) and ClueGO^19^ (**b**). In A, GO Biological Process, Cellular Component, and Molecular Function (blue), pathways (light green), mouse phenotype (yellow), coexpression (pink), disease (dark green), interactions (purple). The full list of gene set enrichment results and P values are in Supplementary Dataset 1. Representative Duodenal *CCNB1* (**d**) stain of and *MKI67* (Ki67, **e**) nuclear immunohistochemistry for Ctl (**c**,**e**, n = 5) and celiac (d, f, n = 5). Ctl (**c**) subject show relatively lower *CCNB1* stain than Celiac (**d**) that correlated with reads per million (RPM) values of 21 and 53 respectively. Ctl (**e**) subject show relatively lower *MKI67* (Ki67) stain than celiac (**f**) that correlated with RPM values of 59 and 157 respectively. Lower images are inlet of the upper images that were recorded at 20x magnification. Scale bar represents 200 and 500 micron.

524 genes showed increased expression in duodenal biopsies from celiac disease patients in comparison to controls (Supplementary Dataset 1). Detailed functional annotation enrichment analyses using ToppGene/ToppCluster and ClueGO^19^ are shown (Fig. 2a,b, and Supplementary Dataset 1). Up-regulated gene signatures were enriched for immune activation including signature for immune response (*P* < 1.42E-13), alpha beta (*P* < 6.55E-55) and gamma delta (*P* < 3.34E-50) T cells, and interferon signaling (*P* < 6.71E-7). In addition, we noted a robust signature enrichment for mitotic cell cycle division (*P* < 2.4E-19), nuclear division (*P* < 7.05E-18), and in the key regulator of cell cycle *CDK1* interactions (*P* < 6.18E-18). Many of those upregulated gene and pathways demonstrate substantial overlap with previous studies (Figures S2, S3, and Supplementary Dataset 1). A substantial number (33/61) of the nuclear division associated genes (GO:0000280) were also significantly differentially expressed in our smaller validation cohort (FDR ≤ 0.05 and fold change ≥1.5, Table S2). Immunohistochemistry confirmed the induction of cyclin B (*CCNB*1), a regulatory protein involved in mitosis, in celiac biopsies in comparison to controls. Furthermore, it demonstrated that the signal for induction of *CCNB1* is noted substantially in the epithelial crypts (Fig. 2c,d). Staining with *Ki-67*, usually used in clinical samples as a marker of cellular proliferation, confirmed a substantial higher nuclear staining in epithelial crypts of celiac patients indicating high proliferative state in epithelia (Fig. 2e,f).

### Mucosal transcriptomics from clinical pathology FFPE tissue can be utilized to correctly classify disease or healthy states in patients undergoing diagnostic endoscopies

**Figure 3:**
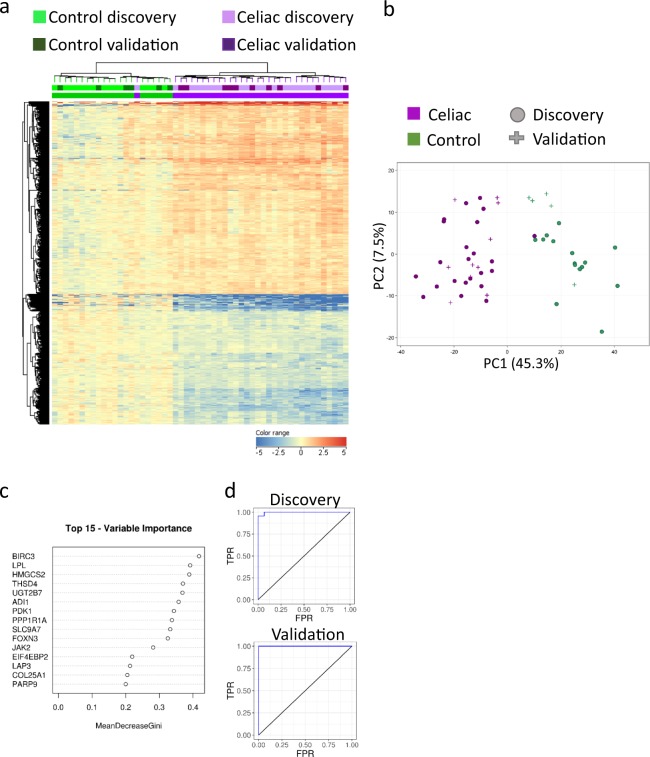
Duodenal transcriptomics can correctly classify disease or healthy states. (**a**) Unsupervised hierarchical clustering of the 878 genes differentially expressed celiac genes is visualized as a heat map for both discovery and validation cohorts with genes up-regulated compared to controls in red and genes down-regulated compared to controls in blue. Above the heat map, individual Control (green) and celiac (purple) are indicated. All Ctl subjects (from the discovery and the independent validation cohorts) cluster in the left branch, and all celiac patients but one cluster in the right branch. (**b**) 878 differentially expressed genes were used to view Ctl (green) and celiac (purple) samples separation of the discovery and validation cohorts on an unsupervised principal component analysis (PCA) plot with nice separation between Ctl and Celiac samples on the PC1 axis. (**c**) Top 15 genes that were prioritized using random forests mean decrease Gini for classification of sample as Ctl or celiac. ROC analysis of the transcriptomic data using random forests classifier in discovery (**d**) and independent validation (**e**) cohorts showing the area under curve (AUC) with high accuracy.

To evaluate the transcriptome ability to correctly classify disease or healthy states we used both unsupervised and supervised approaches. Unsupervised hierarchical clustering using the celiac core 878 genes demonstrated that all discovery Ctl samples grouped in cluster one, while all celiac disease patients but one grouped in cluster two (Fig. 3a). Similarly, all control samples from the independent validation cohort grouped in cluster one and all celiac patients grouped in cluster two (Fig. 3a). Unsupervised Principal Coordinates Analysis (PCA) to view patients’ separation using the 878 core celiac genes and the top two dimensions showed that all control patients are separated from all celiac patients but one that clustered with controls in the discovery and validation cohorts (Fig. 3b), and that the 6 samples that had longer processing time clustered in a similar fashion (Figure S4). Similar unsupervised approaches (PCA and hierarchical clustering) were applied to the 403 genes that were shared between at least 2 transcriptomics datasets with similar results (Figure S2b,c). Consistently, one celiac subject with relatively lower positive anti TTG level (27 U/ml, normal <10 U/ml) tended to cluster closer to controls.

We used transcriptomic-based supervised machine learning approach on the discovery cohort to develop a classification model and then tested the accuracy of the model on the independent validation cohort. A Receiver operating characteristic (ROC) area under the curve (AUC) of 0.97 was obtained when using supervised learning Random Forests (RF) model and all 878 genes in the discovery cohort, and AUC of 1 in the validation cohort (Fig. 3c–e). The genes with the highest contribution to the classification, as calculated by mean decreased gini^21^ were *BIRC3, LPL, HMGCS2, THSD4*, and *UGT2B7* (Fig. 3c). After narrowing the RF to use only those five top contributing genes, the classification improved the ROC AUC to 1 in both discovery and validation cohorts. Using Support Vector Machine (SVM), as another supervised classification algorithm, developed on the discovery cohort and tested on the validation cohort resulted in comparable accuracy of 97.4% and 100% in the discovery and validation cohorts respectively using all genes, with only one celiac sample misclassified as control. Altogether, those results show high accuracy of the transcriptomic data to differentiate celiac from non-celiac control biopsies. Such transcriptomics-based methodology can be applied on suboptimally oriented biopsies to increase accuracy of celiac diagnosis, and if future non-endoscopic sampling devices to obtain duodenal mucosal cells^25^ will be introduced clinically.

### Celiac disease patients exhibit specific increased cell cycle associated signatures not captured in Crohn Disease

**Figure 4:**
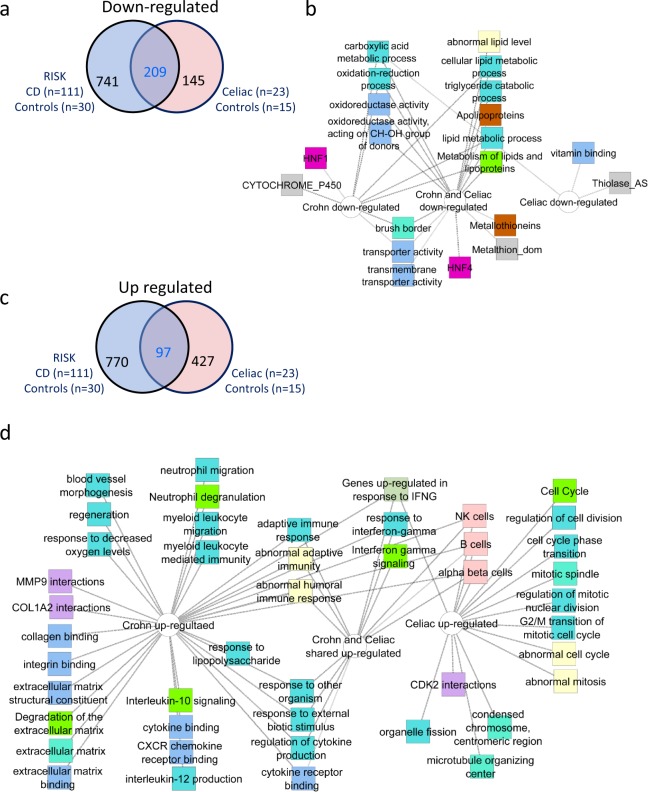
The specific increased cell cycle signatures in celiac disease is not captured in Crohn Disease. (**a**) Venn diagram shows the 209 of the 354 down-regulated celiac genes overlapping the down regulated 950 core RISK Crohn Disease^22,24^ signature (FC ≥1.5, FDR <0.05). (**b**) Functional annotation enrichment analyses of the down-regulated shared and unique genes in **a** using ToppGene^17^, ToppCluster^18^, and Cytoscape^20^ are shown. (**c**) Venn diagram shows the 97 of the 524 up-regulated celiac genes overlapping the up regulated 867 core RISK Crohn Disease^22,24^ signature (FC ≥1.5, FDR <0.05). (**d**) Functional annotation enrichment analyses of the down-regulated shared and unique genes in A using ToppGene^17^, ToppCluster^18^, and Cytoscape^20^ are shown with a celiac-unique cell and nuclear division associated signature. Network nodes: GO Biological Process, Cellular Component, and Molecular Function (blue), pathways (light green), mouse phenotype (yellow), gene family (brown), coexpression (pink), disease (dark green), domain (gray), interactions (purple).

Crohn Disease (CD) is another inflammatory condition that involves the small intestine. We recently characterized the core signature of the inflamed Crohn disease ileum^22–24^. Importantly, a substantial number of genes passed the expression filtering criteria in both studies (see methods). Using a Venn diagram, we show (Fig. 4a and Supplementary Dataset 1) that out of the 354 celiac down regulated genes, 59% (209/354) overlapped with the reduced Crohn signature. Functional annotation enrichments analyses to identify signatures associated with the 741 unique Crohn disease genes, the 209 Crohn/celiac disease shared genes, and the 145 unique Celiac disease genes is shown in Fig. 4b. Remarkable overlap is shown for the Crohn/celiac disease shared reduced signatures including the decrease in epithelial lipid metabolism, oxidoreductase activity, and brush border transport signatures.

In contrast, a significantly smaller proportion [19% (97/427, Chi squares p < 0.001] of the celiac disease 524 up-regulated genes overlapped with the induced Crohn disease signature (Fig. 4c). Functional annotation enrichments analyses were used to identify signature associated with the 770 genes that were induced in Crohn disease, for the 97 shared genes, and for the 427 unique Celiac disease genes (Fig. 4d). While we noted shared enriched signatures for adaptive immune-related pathways and interferon gamma, we also identified more unique Crohn disease associated and Celiac disease associated enriched pathways. The up-regulated Crohn disease signature exhibited more specific enrichments for signatures associated with innate immune pathways and with a strong signal for granulocytes, an extracellular matrix signature, and for CXCR chemokines signaling. In contrast, the enrichment for cell cycle and mitosis was more uniquely represented in the celiac disease up regulated genes.

## Discussion

Using archived clinical FFPE duodenal biopsies and high-throughput transcriptome sequencing of celiac and control subjects we captured many of the previously described pathogenic pathways associated with celiac disease^9–12^, suggesting that our analysis is robust, and that using FFPE clinical samples is a valid approach. We provide evidence for host gene expression profiles driving lymphocyte activation and cytokine signaling in treatment naïve pediatric celiac disease. Our data also suggest a robust induction in epithelial proliferation and nuclear division pathways coupled with reduced mature epithelial metabolic functions in celiac disease, pointing to enhanced proliferative over epithelial differentiation signals. These pathways were validated in our independent celiac sub-cohort, and in recently published celiac disease datasets^9,10^, and defined a celiac disease transcriptomics signature of 403 genes that exhibit differential expression in at least two studies including our own. Novel comparison of the celiac disease core transcriptomic signature with that observed in Crohn disease demonstrated similar and divergent pathways that can shed light on those intestinal inflammatory diseases. Such comparison emphasized the more unique signal of increased proliferation noted in celiac disease. Finally, we show high accuracy of the transcriptomic data to differentiate celiac from non-celiac control biopsies. If future attempts for non-endoscopic sampling device^25^ to obtain duodenal mucosal cells will be successful, such transcriptomic approach can aid in accurate diagnosis of celiac subjects, in conjunction with celiac serology.

We emphasize substantial similarities but also differences associated with Crohn disease and celiac disease pathogenesis. We demonstrate a large overlap of the repressed epithelial mature metabolic signatures in both. However, we noted a substantial divergence of the up-regulated epithelial and immune associated signatures. These differences include an intensified signature linked to innate granulocyte immune responses and extracellular matrix observed more specifically in Crohn disease (Fig. 4d) as opposed to the adaptive immune signature linked to both celiac disease and Crohn disease. It is possible that the adaptive immune response signals the epithelia to divide in leading to crypt hyperplasia in celiac disease, while the innate and extracellular matrix signals oppose such proliferative signals in Crohn Disease. An increased rate of cell production with no significant difference in mitotic duration was noted using microscopic technologies in celiac disease already in the 1970–90s^26–28^. Here we support those observations using an independent molecular transcriptomics and systems biology approach. Multiple inflammatory cytokines (i.e. TNF and Interferon-γ) regulate intestinal epithelial proliferation at the crypt base^29,30^, inducing or restricting intestinal epithelial proliferation and cell death^31,32^ depending on the circumstances. Differences between Crohn disease and celiac disease in this respect may be driven by the role of the gut microbiota that was already linked to Crohn disease pathogenic processes in several large cohorts^33^. The role of microbiota in celiac disease was linked to different metabolic patterns of gluten break down^34^ and was shown to be different in infants with an affected first degree relative^35^, but the overall microbial composition has not yet been fully defined in large human cohorts of celiac disease, and is still controversial^36^.

Our study has several strengths, but also some limitations. Using FFPE clinically archived biopsies and novel analytic approaches we captured many of the previously reported pathways identified in recently published transcriptomics dataset that used research allocated fresh biopsies, supporting the robustness of our methodology and findings. In addition, we show that transcriptomic data of clinically archived samples was able to accurately classify disease or healthy states in both discovery and independent validation cohorts. We emphasize the robust signal of cell proliferation in the transcriptomic data and confirmed its specificity to epithelial crypts by *CCNB1* and *Ki67* immunohistochemistry staining. We used whole biopsies, composed of a mixture of cellular components, rather than single cell transcriptomics. Future studies using single cell preparations, prioritized by the current dataset, will be important for further cellular subset characterizations. However, there are also advantages in using whole biopsies in the clinical setting to capture the overall pathogenic process, and as a potential future diagnostic tool.

In summary, our celiac disease transcriptomics cohort, based on clinically stored FFPE samples, is the largest to date, and was able to identify important molecular pathogenic signatures emphasizing a signal for epithelial proliferation over differentiation, coupled with increased adaptive immune signature. We validate those in recently published independent cohorts^10^ and in our validation dataset. We highlight important biologic differences between Crohn disease and celiac disease, two inflammatory conditions known to cause small intestine inflammation with a more intensified signature of innate granulocytes activation linked to Crohn disease and a more specific epithelial proliferative signature in celiac disease. Integrating this knowledge from transcriptomics datasets paves the way to more mechanistic studies that altogether will lead to new insights regarding pathogenesis of both diseases, and to future molecular-based prevention and therapies for those chronic conditions.

## Supplementary information

Supplementary information

Dataset 1

## Data Availability

Duodenal mRNAseq data sets were deposited into GEO [GSE131705].

## References

[1] Fasano A, Catassi C (2012). Clinical practice. Celiac disease. The New England journal of medicine.

[2] Green PH, Cellier C (2007). Celiac disease. The New England journal of medicine.

[3] Szajewska H (2016). Gluten Introduction and the Risk of Coeliac Disease: A Position Paper by the European Society for Pediatric Gastroenterology, Hepatology, and Nutrition. Journal of pediatric gastroenterology and nutrition.

[4] Rubio-Tapia A (2009). Increased prevalence and mortality in undiagnosed celiac disease. Gastroenterology.

[5] Catassi C (2010). Natural history of celiac disease autoimmunity in a USA cohort followed since 1974. Annals of medicine.

[6] Lohi S (2007). Increasing prevalence of coeliac disease over time. Alimentary pharmacology & therapeutics.

[7] Lebwohl B, Sanders DS, Green PHR (2018). Coeliac disease. Lancet.

[8] Hedegaard J (2014). Next-generation sequencing of RNA and DNA isolated from paired fresh-frozen and formalin-fixed paraffin-embedded samples of human cancer and normal tissue. PloS one.

[9] Leonard MM (2019). RNA sequencing of intestinal mucosa reveals novel pathways functionally linked to celiac disease pathogenesis. PloS one.

[10] Bragde H, Jansson U, Fredrikson M, Grodzinsky E, Soderman J (2018). Celiac disease biomarkers identified by transcriptome analysis of small intestinal biopsies. Cellular and molecular life sciences: CMLS.

[11] Diosdado B (2004). A microarray screen for novel candidate genes in coeliac disease pathogenesis. Gut.

[12] Acharya P (2018). First Degree Relatives of Patients with Celiac Disease Harbour an Intestinal Transcriptomic Signature that Might Protect them from Enterocyte Damage. Clinical and translational gastroenterology.

[13] Scanlon SA, Murray JA (2011). Update on celiac disease - etiology, differential diagnosis, drug targets, and management advances. Clinical and experimental gastroenterology.

[14] Tuerk A, Wiktorin G, Guler S (2017). Mixture models reveal multiple positional bias types in RNA-Seq data and lead to accurate transcript concentration estimates. PLoS Comput Biol.

[15] Haberman Y (2019). Ulcerative colitis mucosal transcriptomes reveal mitochondriopathy and personalized mechanisms underlying disease severity and treatment response. Nature communications.

[16] Bray NL, Pimentel H, Melsted P, Pachter L (2016). Near-optimal probabilistic RNA-seq quantification. Nature biotechnology.

[17] Chen J, Bardes EE, Aronow BJ, Jegga AG (2009). ToppGene Suite for gene list enrichment analysis and candidate gene prioritization. Nucleic acids research.

[18] Kaimal V, Bardes EE, Tabar SC, Jegga AG, Aronow BJ (2010). ToppCluster: a multiple gene list feature analyzer for comparative enrichment clustering and network-based dissection of biological systems. Nucleic acids research.

[19] Bindea G (2009). ClueGO: a Cytoscape plug-in to decipher functionally grouped gene ontology and pathway annotation networks. Bioinformatics.

[20] Saito R (2012). A travel guide to Cytoscape plugins. Nature methods.

[21] Svetnik V (2003). Random forest: a classification and regression tool for compound classification and QSAR modeling. J Chem Inf Comput Sci.

[22] Haberman Y (2018). Long ncRNA Landscape in the Ileum of Treatment-Naive Early-Onset Crohn Disease. Inflammatory bowel diseases.

[23] Haberman, Y. *et al*. Age-of-diagnosis dependent ileal immune intensification and reduced alpha-defensin in older versus younger pediatric Crohn Disease patients despite already established dysbiosis. *Mucosal immunology*, 10.1038/s41385-018-0114-4 (2018). 10.1038/s41385-018-0114-4 PMC6375755 30542108

[24] Haberman Y (2014). Pediatric Crohn disease patients exhibit specific ileal transcriptome and microbiome signature. The Journal of clinical investigation.

[25] Otuya DO (2018). Non-endoscopic biopsy techniques: a review. Expert review of gastroenterology & hepatology.

[26] Savidge TC, Walker-Smith JA, Phillips AD (1995). Novel insights into human intestinal epithelial cell proliferation in health and disease using confocal microscopy. Gut.

[27] Wright N, Watson A, Morley A, Appleton D, Marks J (1973). Cell kinetics in flat (avillous) mucosa of the human small intestine. Gut.

[28] Wright N (1973). The cell cycle time in the flat (avillous) mucosa of the human small intestine. Gut.

[29] Andrews C, McLean MH, Durum SK (2018). Cytokine Tuning of Intestinal Epithelial Function. Frontiers in immunology.

[30] Lindemans CA (2015). Interleukin-22 promotes intestinal-stem-cell-mediated epithelial regeneration. Nature.

[31] Bradford EM (2017). Epithelial TNF Receptor Signaling Promotes Mucosal Repair in Inflammatory Bowel Disease. J Immunol.

[32] Nava P (2010). Interferon-gamma regulates intestinal epithelial homeostasis through converging beta-catenin signaling pathways. Immunity.

[33] Braun T (2019). Individualized Dynamics in the Gut Microbiota Precede Crohn’s Disease Flares. The American journal of gastroenterology.

[34] Caminero A (2016). Duodenal Bacteria From Patients With Celiac Disease and Healthy Subjects Distinctly Affect Gluten Breakdown and Immunogenicity. Gastroenterology.

[35] Olivares M (2018). Gut microbiota trajectory in early life may predict development of celiac disease. Microbiome.

[36] de Meij TG (2013). Composition and diversity of the duodenal mucosa-associated microbiome in children with untreated coeliac disease. Scandinavian journal of gastroenterology.

