# Supplementary material for: Defining the Celiac Disease Transcriptome using Clinical Pathology Specimens Reveals Biologic Pathways and Supports Diagnosis
Source: Sci Rep. 2019 Nov 7;9:16163. doi: 10.1038/s41598-019-52733-1 (PMC6838157; doi:10.1038/s41598-019-52733-1)
Supplement: Supplementary file 1 — Supplementary information [file 41598_2019_52733_MOESM1_ESM.docx]

**Defining the Celiac Disease Transcriptome using Clinical Pathology Specimens Reveals Biologic Pathways and Supports Diagnosis**

**Short title:** Transcriptomic Signature of Celiac disease

Nurit Loberman-Nachum, MD^1,2,3^ , Katya Sosnovski, MS^1,3^, Ayelet Di Segni, PhD^1^, Gilat Efroni, PhD^1^, Tzipi Braun, MS^1^, Marina BenShoshan, MS^1,3^ Lait Anafi, MS^4^, Camila Avivi, PhD^4^, Iris Barshack, MD^3,4^, Dror S Shouval MD^1,3^, Lee A Denson^5^, MD, Amnon Amir, PhD^1,3^, Ron Unger, PhD^2^, Batia Weiss, MD ^1,3^ Yael Haberman, MD, PhD^1,2,5^*

^1^The Pediatric Gastroenterology Unit, The Edmond and Lily Safra Children’s Hospital, Sheba Medical Center, Tel-Hashomer, Israel

^2^Mina and Everard Goodman Faculty of Life Science, Bar-Ilan University, Ramat Gan, Israel

^3^Sackler Faculty of Medicine, Tel Aviv University, Tel Aviv, Israel.

^4^Institute of Pathology, Sheba Medical Center, Tel Hashomer, Israel.

^5^Cincinnati Children’s Hospital Medical Center, Department of Pediatrics, University of Cincinnati College of Medicine, Cincinnati, OH, USA.

***Corresponding Author:**

Yael Haberman, MD, PhD

Pediatric Gastroenterology, Hepatology & Nutrition Unit

The Edmond and Lily Safra Children’s Hospital, Sheba Medical Center, Tel Hashomer, Israel

Telephone number: 972-3-5302692

[Yael.Haberman@sheba.health.gov.il](mailto:Yael.Haberman@sheba.health.gov.il) and [Yael.haberman@cchmc.org](mailto:Yael.haberman@cchmc.org)

**Supplemental Materials**

**Supplemental tables**

**Table S1** (page 3). Summary of celiac transcriptomic datasets (above 10 celiac subjects) and their use for validation of the current study results

**Table S2** (page 4). Nuclear division associated genes (n-33) expression mean read per million (RPM) levels in discovery and the validation cohorts of across controls and celiac subjects.

**Table S3** (page 5). Primers used in qPCR analyses

**Supplementary Dataset 1 (**separate excel file**)**. The core 878 genes, and functional annotation enrichments analyses (see additional spreadsheet), and the 403 differentially expressed genes between celiac and Ctl in 2 of 3 studies (current study, Bragde et al[^1^](#_ENREF_1) and Leonard et al[^2^](#_ENREF_2))

**Supplemental Figures**

**Figure S1** (page 6)**.** Scattered plot demonstrating Lexogen QuantSeq 3' mRNAseq log2 transformed Reads per Million (RPM) of paired FFPE and fresh samples.

**Figure S2** (page 7). Validation of the core celiac signature using the 403 differentially expressed genes between celiac and Ctl in 2 of 3 studies (current study, Bragde et al[^1^](#_ENREF_1) and Leonard et al[^2^](#_ENREF_2)).

**Figure S3** (page 8). Validation of the core celiac signature in an independent recently published Bragde et al[^1^](#_ENREF_1) data, using ToppCluster ^[3](#_ENREF_3)^, and Cytoscape [^4^](#_ENREF_4) platforms.

**Figure S4** (page 9)**.** 878 differentially expressed genes were used to view Ctl (green) and celiac (purple) samples separation of the samples archived during (n=48) or before (n=6) 2017.

**Table S1.** Summary Celiac intestinal high throughput transcriptomic datasets with above 10 celiac subjects and the Crohn Disease study used for comparison.

| Transcriptomic Study | Colon samples # | Publication | Platform | Method | Deposition | Used here for discovery/validation |
| --- | --- | --- | --- | --- | --- | --- |
| Pediatric celiac and control | 33 celiac  21 controls | Current paper | RNAseq | Illumina using Lexogen QuantSeq 3' mRNAseq | GSE131705 | Yes. Original analyses. |
| RISK pediatric IBD inception | 139 CD  38 controls | Current paper. Haberman et al, JCI 2014[^5^](#_ENREF_5) & IBD J 2018[^6^](#_ENREF_6) | RNAseq | Illumina TruSeq mRNAseq | GSE117993 | Yes, to compare with small intestine Crohn signature |
| Adult and pediatric Celiac | 15 celiac  7 controls | Diosdado B. et al. Gut, 2004[^7^](#_ENREF_7) | Microarray | Gene microarray from the University Health Network of Toronto | EBI: E-MEXP-42 | Yes, using processed dataset |
| Adult celiac and first degree relatives | 12 celiac  12 first-degree relatives (FDR)  12 controls | Acharya et al. Clinical and Translational Gastroenterology, 2018[^8^](#_ENREF_8) | Microarray | Illumina BeadChips | Not available |  |
| Pediatric celiac and control | 20 celiac  20 controls | Bragde H et al. Cellular and Molecular Life Sciences, 2018[^1^](#_ENREF_1) | RNAseq | TruSeq Stranded Total RNA | Not available | Yes, using processed dataset |
| Adult celiac and controls | 12 active celiac  15 controls | Leonard MM et al  PlosOne, 2019[^2^](#_ENREF_2) | RNAseq | KAPA stranded mRNA-Seq | PRJNA528755 | Yes, using raw files |

**Table S2:** Nuclear division associated genes (n-33) expression mean read per million (RPM) levels in training and the validation cohorts of across controls and celiac subjects

| Gene Symbol | Gene Name | FC Celiac vs non-Celiac training | Celiac vs non-Celia corrected P | FC Celiac vs non-Celiac validation | Celiac vs non-Celia corrected P |
| --- | --- | --- | --- | --- | --- |
| ASPM | abnormal spindle microtubule assembly | 2.22 | 0.00005 | 1.55 | 0.04164 |
| AURKA | aurora kinase A | 2.13 | <0.00001 | 2.04 | 0.00532 |
| BIRC3 | baculoviral IAP repeat containing 3 | 2.56 | <0.00001 | 1.98 | 0.00164 |
| BIRC5 | baculoviral IAP repeat containing 5 | 1.55 | 0.00079 | 1.60 | 0.00410 |
| BRCA2 | BRCA2, DNA repair associated | 1.82 | 0.00181 | 1.62 | 0.00158 |
| CCNA2 | cyclin A2 | 1.63 | 0.00027 | 1.77 | 0.03460 |
| CCNB1 | cyclin B1 | 1.99 | 0.00008 | 2.53 | 0.00048 |
| CDC6 | cell division cycle 6 | 1.90 | 0.00007 | 2.70 | 0.00026 |
| CDCA2 | cell division cycle associated 2 | 2.44 | 0.00015 | 2.68 | 0.00609 |
| CDCA8 | cell division cycle associated 8 | 1.83 | 0.00116 | 1.61 | 0.04078 |
| CEP55 | centrosomal protein 55 | 3.64 | <0.00001 | 2.49 | 0.00630 |
| DLGAP5 | DLG associated protein 5 | 2.897508 | 0.000178 | 1.69 | 0.04796 |
| HELLS | helicase, lymphoid specific | 1.73 | 0.00010 | 1.62 | 0.00421 |
| KIF14 | kinesin family member 14 | 2.04 | 0.00024 | 1.73 | 0.00926 |
| KIF15 | kinesin family member 15 | 2.17 | 0.00046 | 1.83 | 0.01711 |
| KIF4A | kinesin family member 4A | 2.40 | 0.00012 | 2.14 | 0.01155 |
| KNTC1 | kinetochore associated 1 | 1.63 | 0.00148 | 1.62 | 0.00333 |
| MKI67 | marker of proliferation Ki-67 | 2.12 | <0.00001 | 1.67 | 0.00643 |
| NCAPG | non-SMC condensin I complex subunit G | 2.15 | 0.00296 | 3.83 | 0.00410 |
| NDC80 | NDC80 kinetochore complex component | 2.132747 | 0.002079 | 1.63 | 0.04910 |
| NEK2 | NIMA related kinase 2 | 1.56 | 0.00061 | 1.77 | 0.01204 |
| NUF2 | NUF2, NDC80 kinetochore complex component | 2.14 | 0.00069 | 2.25 | 0.00668 |
| PLK1 | polo like kinase 1 | 1.89 | 0.00003 | 2.18 | 0.00014 |
| PRC1 | protein regulator of cytokinesis 1 | 2.22 | 0.00004 | 1.94 | 0.02114 |
| RANBP1 | RAN binding protein 1 | 1.61 | 0.00004 | 1.68 | 0.00321 |
| SGO2 | shugoshin 2 | 1.72 | 0.00058 | 1.62 | 0.01119 |
| SMC2 | structural maintenance of chromosomes 2 | 1.62 | 0.00003 | 1.62 | 0.00249 |
| SPDL1 | spindle apparatus coiled-coil protein 1 | 1.74 | 0.00569 | 1.91 | 0.00939 |
| TOP2A | DNA topoisomerase II alpha | 1.68 | 0.00148 | 1.64 | 0.00205 |
| TPX2 | TPX2, microtubule nucleation factor | 2.39 | <0.00001 | 1.95 | 0.00991 |
| TTYH1 | tweety family member 1 | 1.50 | 0.00745 | 1.62 | 0.03355 |
| UBE2S | ubiquitin conjugating enzyme E2 S | 1.81 | 0.00012 | 2.34 | 0.00151 |
| ZFP42 | ZFP42 zinc finger protein | 1.66 | 0.00052 | 1.79 | 0.01704 |

**Table S3**: Primers used in qPCR analyses

*SI* (F:5'-CTGCATTTGAAAGAGGACAGC-3', R:5'ACTCTGCTGTGGAAGTCCTGA-3')

*APOA1* (F:5'-AACAGCTAAACCTAAAGCTCCT-3', R:5'-CAGAACTCCTGGGTCACA-3')

GAPDH (F: 5'-TGGACCTCATGGCCCACA-3', R: 5'-TCAAGGGGTCTACATGGCAA-3')

**Supplemental Figures**

**
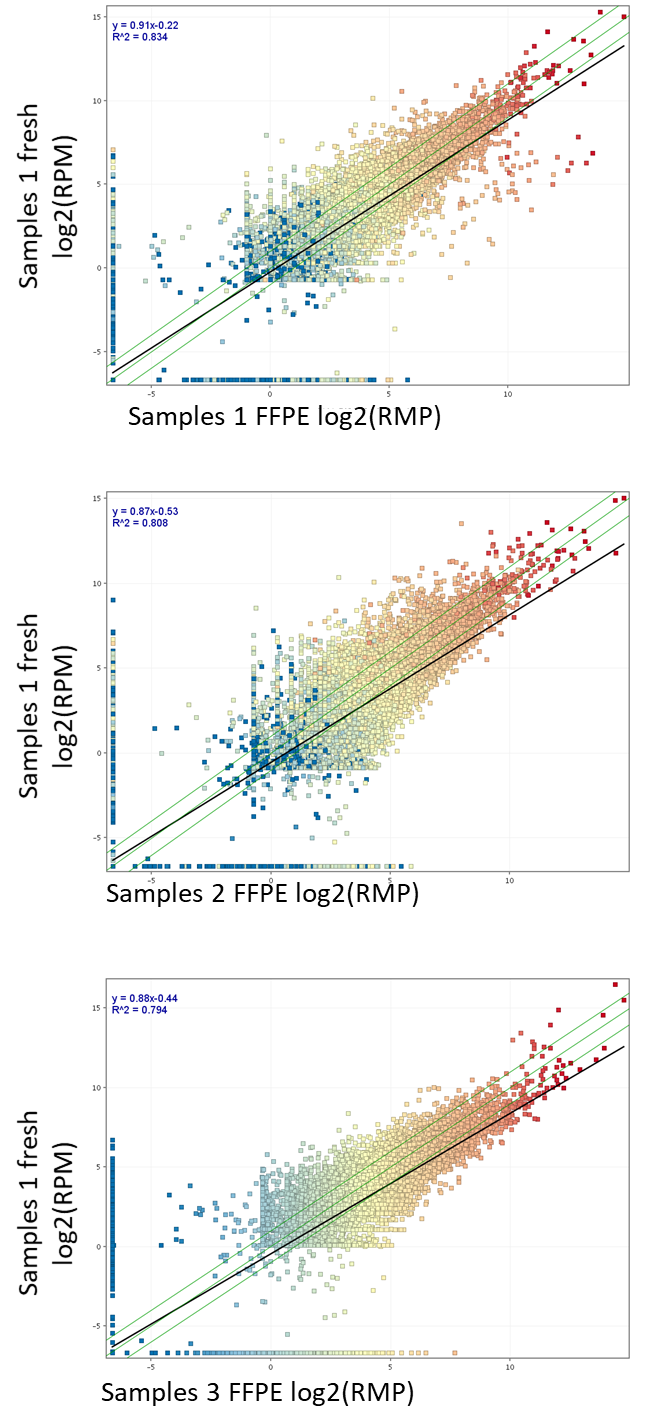
**

**Figure S1.** Scattered plot demonstrating Lexogen QuantSeq 3' mRNAseq log2 transformed Reads per Million (RPM) of paired FFPE and fresh samples obtained from the same rectal biopsy location and processed in a similar pipeline (see methods). Linear correlation and the R^^2^is present on the top left.

**
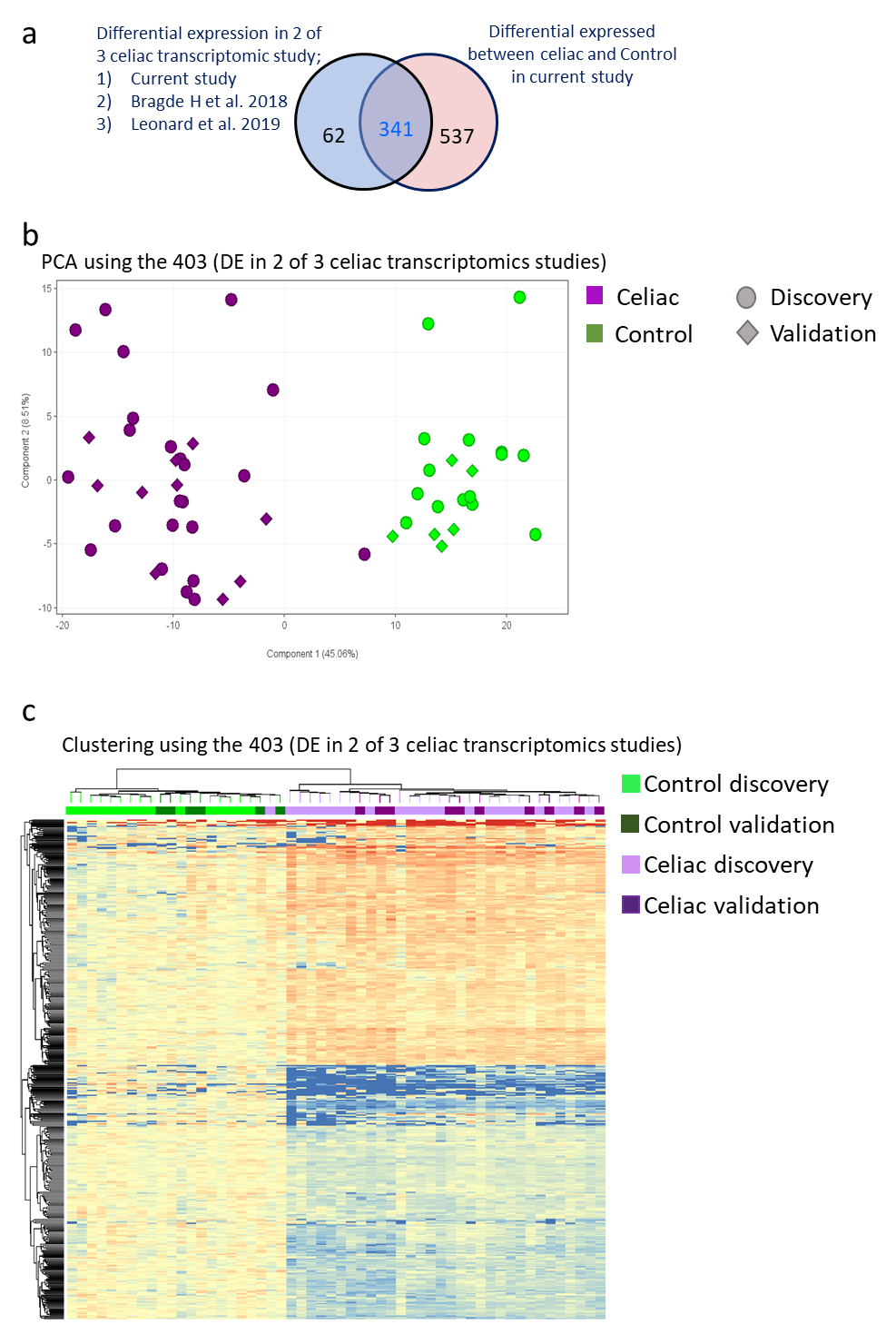
**

**Figure S2**. Validation of the core celiac signature using the 403 differentially expressed genes between celiac and Ctl in 2 of 3 studies (current study, Bragde et al[^1^](#_ENREF_1) and Leonard et al[^2^](#_ENREF_2)). **A**. Venn diagram showing the overlap between those 403 and the current study 878 differentially expressed genes. PCA (**B**) and hierarchical clustering (**C**) using the 403 genes and the current study gene expression matrix and patients.

**
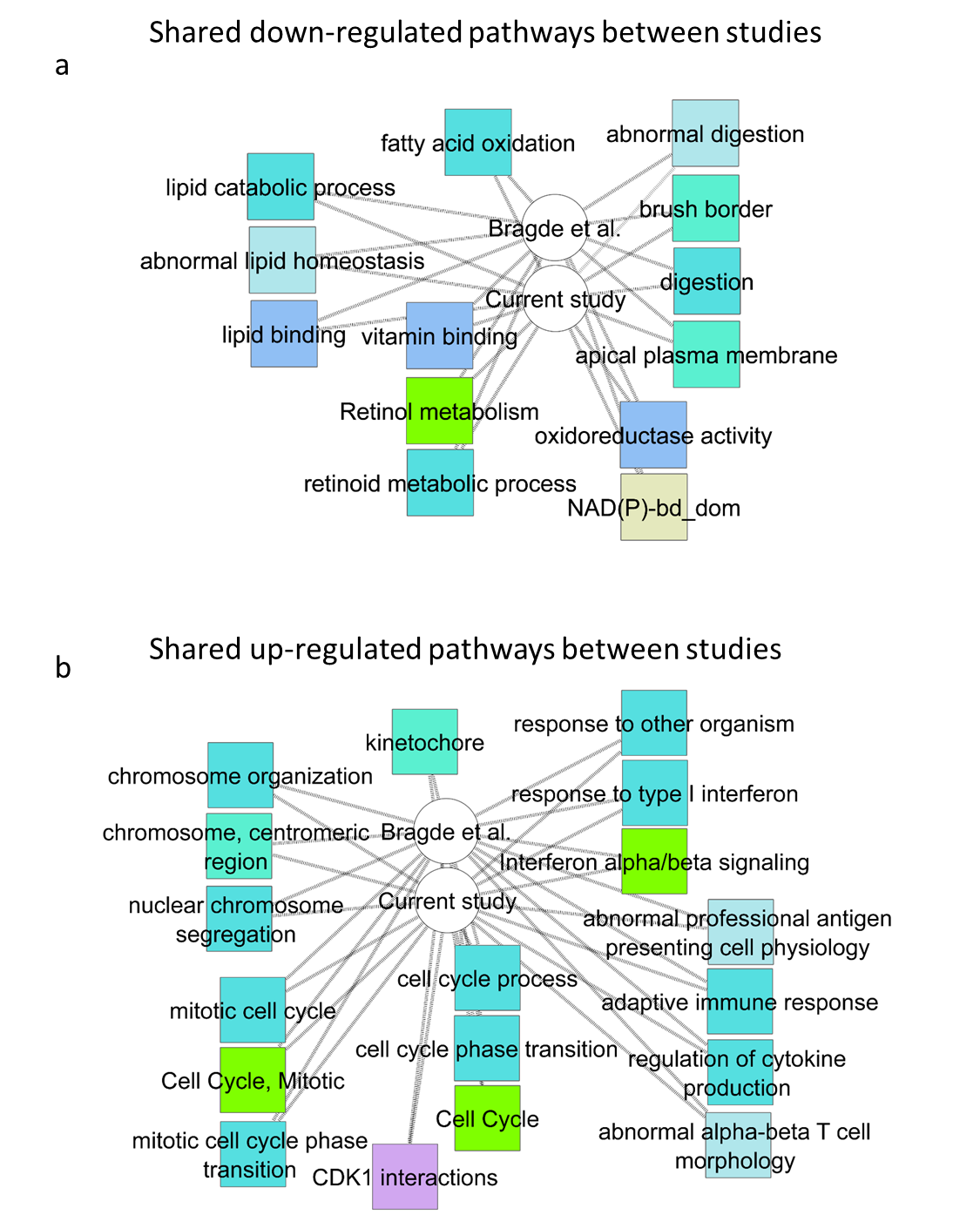
**

**Figure S3**. Validation of the core celiac signature in an independent recently published Bragde et al[^1^](#_ENREF_1) data, using ToppCluster [^3^](#_ENREF_3), and Cytoscape [^4^](#_ENREF_4) platforms.

**
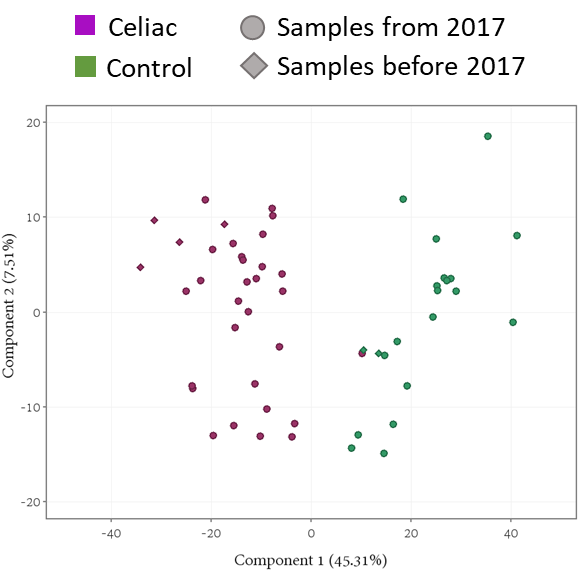
**

**Figure S4.** 878 differentially expressed genes were used to view Ctl (green) and celiac (purple) samples separation of the samples archived during (n=48) or before (n=6) 2017 on an unsupervised principal component analysis (PCA) plot.

**References**

1 Bragde, H., Jansson, U., Fredrikson, M., Grodzinsky, E. & Soderman, J. Celiac disease biomarkers identified by transcriptome analysis of small intestinal biopsies. *Cellular and molecular life sciences : CMLS* **75**, 4385-4401, doi:10.1007/s00018-018-2898-5 (2018).

2 Leonard, M. M. *et al.* RNA sequencing of intestinal mucosa reveals novel pathways functionally linked to celiac disease pathogenesis. *PloS one* **14**, e0215132, doi:10.1371/journal.pone.0215132 (2019).

3 Kaimal, V., Bardes, E. E., Tabar, S. C., Jegga, A. G. & Aronow, B. J. ToppCluster: a multiple gene list feature analyzer for comparative enrichment clustering and network-based dissection of biological systems. *Nucleic acids research* **38**, W96-102, doi:10.1093/nar/gkq418 (2010).

4 Saito, R. *et al.* A travel guide to Cytoscape plugins. *Nature methods* **9**, 1069-1076, doi:10.1038/nmeth.2212 (2012).

5 Haberman, Y. *et al.* Pediatric Crohn disease patients exhibit specific ileal transcriptome and microbiome signature. *The Journal of clinical investigation* **124**, 3617-3633, doi:10.1172/JCI75436 (2014).

6 Haberman, Y. *et al.* Long ncRNA Landscape in the Ileum of Treatment-Naive Early-Onset Crohn Disease. *Inflammatory bowel diseases* **24**, 346-360, doi:10.1093/ibd/izx013 (2018).

7 Diosdado, B. *et al.* A microarray screen for novel candidate genes in coeliac disease pathogenesis. *Gut* **53**, 944-951 (2004).

8 Acharya, P. *et al.* First Degree Relatives of Patients with Celiac Disease Harbour an Intestinal Transcriptomic Signature that Might Protect them from Enterocyte Damage. *Clinical and translational gastroenterology* **9**, 195, doi:10.1038/s41424-018-0059-7 (2018).
